# 

**Published:** 1902-05-10

**Authors:** 


### NOTICE TO CORRESPONDENTS.

All MSS., Letters, Books for Review, and other matters intended for the Editor should be addressed The Editor, "The Hospital," The Hospital Building, 28 & 29 Southampton Street, Strand, W.C.

The Editor cannot undertake to return rejected MS., even when accompanied by stamped directed envelope.

**Notice to Advertisers and Subscribers.**

All Advertisements, Orders for Copies of The Hospital, and Business Communications should be addressed to **THE MANAGER, (Not to the Editor)**, The Hospital, 28 & 29 Southampton Street, Strand, London, W.C.

The Cost of Subscription, post free, is as follows.—Per week, 2½d.; per quarter, 2s. 8d; per half-year, 5s. 3d.; per annum, 10s. 6d. Foreign:—Per annum, 15s. 2d., payable, in all cases, in advance.

**NOTICE.—Vol. XXXI. of "The Hospital" (October 1901, to March 1902), in handsome cloth binding will shortly be on sale at the office of this Journal, price, 7s. 6d. Cases for binding the Numbers contained in this Volume 1s. 6d. Index to Vol. XXXI., 2½d., post free.**

**The Monthly Part for May is now ready. Price 6d., by post 8d.**

### BOOKS RECEIVED.

H. K. Lewis.

"A Concise History of Small-pox and Vaccination in Europe." By Edward J. Edwardes, M.D.Lond.

The Sanitary Publishing Co.

"The Prevention of Infection in Public Vehicles." By Alfred Greenwood, M.D., Ch.B., D.P.H.

E. Merck.

"Annual Report on the Year 1901."

Longmans, Green, and Co.

"The Elements of Mind." By H. Jamyn Brooks.
"Tommy Cornstalk." By J. H. M. Abbott.

### SCRAPS AND GLEANINGS.

A PING-PONG tournament was held recently at Upton House, Tetbury, and the proceeds were given to the local Cottage Hospital.

SIR PETER WALKER, Bart., has presented to the Derbyshire Royal Infirmary, as a memorial of the late Queen Victoria, a complete apparatus for the treatment of lupus patients by the Finsen light system.

THE directors of the Alhambra have granted the use of their theatre for a matinée on May 29th, on behalf of the Lifeboat Saturday Fund. T.R.H. the Prince and Princess of Wales have promised to be present.

THE annual report of the South Devon and East Cornwall Hospital shows that the expenditure during the past year has exceeded the income by £253. At the recent meeting the chairman appealed for more financial assistance from people residing in the West of England.

To enable the foundation stone of the building of the proposed Victoria Memorial Cottage Hospital for Leatherhead to be laid during Coronation week, as is desired, it is necessary that a sum of between £700 and £800 should be raised by that date, so as to complete the amount of £1,600 required.

A FINAL meeting to dispose of the business in connection with the Soldiers' Convalescent Home, Fairfield, Manchester, was recently held. A *résumé* of the work done by the institution since its inception was given, which showed that 145 men had passed through the home during the 20 months it had been opened. A sum of £700 had been raised initially to enable the home to be carried on, and of that sum an amount of £44 remained in the bank, after paying all the accounts. It was decided that this sum should be divided equally between the Ashton Reservists Fund and the Manchester, Salford, and District War Fund.

The two consulting-rooms which have been built at the Salisbury Infirmary as a memorial to the late Dr. Fawson Lee were recently formally opened and handed over to the governors of the institution. The late Dr. Fawson Lee was honorary physician to the Salisbury Infirmary from 1874 to 1899.

The annual general report of the Gateshead Children's Hospital states that £761 has been received towards endowing a cot in memory of Queen Victoria. There has been a slight decrease in the amount of general subscriptions, but in addition to these £104 odd has been received from church collections, workshops, etc.

### THE STUDENT OF MEDICINE.

### APPOINTMENTS.

**Daniel Brough**, M.B., C.M.Edin., L.R.C.P.Lond., M.R.C.S., appointed Deputy Medical Officer of Health for the Borough of Devonport. **H. Brown**, M.B., Ch.B.Vict., appointed Resident Obstetric Officer to the General Infirmary, Leeds. **A. E. N. Browne**, L.R.C.P.&S.Edin., appointed Acting Visiting Surgeon, *pro tem.*, to the Gaol at Wagga Wagga, N.S.W. **W. Clarkson**, L.R.C.P.Edin., L.F.P.S.Glasg., appointed Medical Officer of Health to the Morpeth District Council. **H. Dodgson**, M.B., Ch.B.Edin., appointed Senior Assistant Medical Officer to the Cumberland and Westmorland Asylum. **J. B. Elder**, M.B., Ch..B.Aberd., appointed Junior Assistant Medical Officer to the Cumberland and Westmorland Asylum. **M. F. Gamble**, L.R.C.S.Edin., appointed Medical Superintendent, *pro tem.*, of the Ballarat Lunatic Asylum, Victoria, N.S.W. **W. W. Hearne**, M.B.Vict., appointed Public Vaccinator, *pro tem.*, for the North-Eastern Vaccination District of Victoria, N.S.W. **Charles Albert Hingston**, M.D., B.Sc. Lond., M.R.C.S., appointed Honorary Physician to the Crownhill Convalescent Home, Plymouth. **Andrew Honman**, M.R.C.S., appointed Certifying Medical Practitioner for the purposes of the Factories and Shop Acts at Williamstown, Victoria, N.S.W. **Herbert Jones**, L.R.C.S.Irel., L.S.A., appointed Medical Officer of Health for the Bromyard Urban District. **Andrew J. Laird**, M.D., D.P.H., Assistant Medical Officer of Health for the County of Lanark, appointed Medical Officer of Health for the Borough of Crewe. **Arthur Wharton Lemarchand**, L.R.C.P.Lond., M.R.C.S., appointed Honorary Medical Officer to the North Devon Infirmary, Barnstaple. **Peter Lynch**, L.R.C.P., L.R.C.S.Edin., appointed Certifying Medical Practitioner for the purposes of the Factory and Shop Acts at Carlton, Victoria, N.S.W. **Charles Herbert Melland**, M.D.Lond., M.R.C.P., appointed Honorary Physician to the Ancoats Hospital, Manchester. **W. C. C. Muir**, M.B.Glasg., appointed Medical Officer of Health for the Shire of Alberton, Victoria, N.S.W. **W. L. Mullen**, M.D.Melb., appointed Medical Superintendent, *pro tem.*, to the Yarra Bend Lunatic Asylum, Victoria, N.S.W. **J. S. Park**, L.R.C.P.Lond., appointed Officer of Health for the Shire of Cranbourne, Victoria, N.S.W. **F. Peipers**, M.D.Berl., appointed Officer of Health for the Shire of Kerong, Victoria, N.S.W. **Robert Stevenson**, M.D., appointed Medical Officer of Health to the Bootle Postal District. **Sydney G. Tippett**, M.B.Lond., appointed Assistant Surgeon to St. Bartholomew's Hospital, Chatham. **A. F. Turner**, L.R.C.P., L.R.C.S.Edin., reappointed Medical Officer of Health to the Tewkesbury Corporation. **J. Sim Wallace**, M.D., D.Sc., L.D.S.Eng., appointed Honorary Dental Surgeon to the West End Hospital for Diseases of the Nervous System. **R. T. Williamson**, M.D., F.R.C.P.Lond., appointed Honorary Assistant Physician to the Manchester Royal Infirmary.

### "The Hospital" Institutional Library.

"Hospitals and Asylums of the World." 4 Vols., with a Portfolio of Plans, complete £12 12s.

"Burdett's Hospitals and Charities, 1902." 5s.

"Cottage Hospitals, General, Fever, and Convalescent." 10s. 6d.

"Hospital Expenditure: The Commissariat." 2s. 6d.

"A Legal Handbook for the Use of Hospital Authorities." 2s. 6d.

"The Cottage Hospital Case Book or Register of Patients." 10s. and 12s. 6d.

"The Furnishing and Appliances of a Cottage Hospital." 6d.

All these are published by the Scientific Press, Ltd., and may be obtained through any bookseller, or direct from the publishers, 28 and 29 Southampton Street, Strand, London, W.C.

